# Deep learning–driven image captioning: Progress through transformers and large language models

**DOI:** 10.1371/journal.pone.0345012

**Published:** 2026-03-16

**Authors:** Priyanka Panchal, Vishal Polara, Siddaraj U, Abdullah Baz, Shobhit K. Patel

**Affiliations:** 1 Department of Information Technology, Madhuben and Bhanubhai Patel Institute of Technology, The Charutar Vidya Mandal (CVM) University, New Vallabh Vidya Nagar, Gujarat, India; 2 Department of Computer Engineering-AI and BD, Marwadi University, Rajkot, Gujarat, India; 3 Manipal Institute of Technology, Manipal Academy of Higher Education, Manipal, India; 4 Department of Computer and Network Engineering, College of Computing, Umm Al-Qura University, Makkah, Saudi Arabia; Bayer Crop Science United States: Bayer CropScience LP, UNITED STATES OF AMERICA

## Abstract

This paper provides a novel deep learning model for captioning of images by using an advanced vision transformer architecture with a powerful LLM. Proposed models show a significant improvement over traditional CNN-RNN hybrids and existing transformer-based approaches by integrating a unique cross-attention mechanism that enables deep alignment between linguistic context and visual features. We show the superiority of our proposed architecture through extensive evaluation on different datasets like MSCOCO, Flickr30K, and NoCaps. The proposed model consistently shows good performance for leading methods such as GIT, BLIP-2, and CoCa across a comprehensive suite of metrics. On the MS COCO dataset, the BLEU-4, METEOR, and CIDEr scores of proposed models are equal to 0.495, 0.390, and 1.32, respectively. In this paper, we have critically analyzed the key challenges of this field, like enhancing caption diversity, ensuring robust multimodal alignment, and mitigating inherent biases. By providing a new performance level, the proposed model provides a source of reference for the next generation of image captioning systems. The results show the efficiency of our fusion strategy and facilitate the development of techniques that use models that can produce more precise, contextually rich, and human-like image depictions. This work supports SDG 9 (Industry, Innovation, and Infrastructure) by advancing multimodal AI systems, and SDG 4 (Quality Education) by enabling intelligent and accessible image understanding technologies.

## 1. Introduction

The main goal of image captioning is to generate a natural language description of given visual content automatically, which is a good task in the field of artificial intelligence. It works as a critical intersection point between NLP and computer vision, which requires the machines to be built in a way that not only provides objects and scenes in an image but also provides their most important aspects in an effective and linguistically meaningful manner. This capacity is the basis for a deep comprehension of visual scenes, and the increasing proliferation of images on the internet has further boosted the demand for effective and automated image annotation techniques [1].

**Fig 1:**
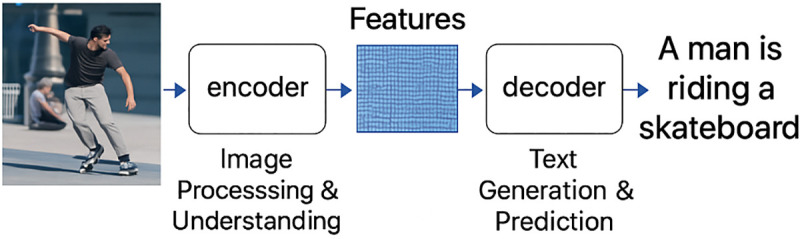
Encoder-decoder model for image captioning.

In the past few years, image captioning methods have become more popular. Previous methods, which depend on template-based and retrieval-based strategies, provide a way to have more powerful neural network-based models. A significant leap forward was created with the encoder-decoder framework (Fig 1) that typically uses CNN for extracting visual features and RNN to generate descriptive text. The transformer architecture has surfaces as a dominant force and leverages a multi-head attention mechanism, which appeared as an effective way to capture intricate relationships between various elements of an image and the words in a sentence [2]. By having these advancements, the introduction of LLM has also added to enhance the capabilities of captioning systems, which enhance their extensive understanding of language to produce more correct, consistent, and meaningful captions [3,4].

When these advanced features have increased the boundaries of performance still there is still a need for models that can achieve a more powerful and nuanced compatibility between image and word domains. To remove this, in this paper, we present a novel image captioning model that specifically uses an advanced transformer-based vision encoder with a powerful LLM decoder [5,6]. Our proposed Model architecture is designed to optimise the flow of information between modalities, leading to a superior understanding of visual context and more precise language generation. We show that the proposed implementation of new state-of-the-art results is achieved in terms of the model, outperforming existing leading models such as BLIP-2, GIT, and CoCa across multiple benchmark datasets, including MS COCO, Flickr30K, and NoCaps. The paper describes the architecture of our model, the assessment of its performance in significant detail, and evaluates its coverage contributions to the future of research into image captioning, and specifically, key issues of the diversity of captions and multimodal alignment [7].

The introduction of large language models (LLMs) has further increased the potential of image captioning potential even further, based on what it has already achieved [8–10]. LLMs are pre-trained using vast amounts of textual information and can generate more precise, coherent, and contextualized texts [11]. The current research paper will explore the history of image captioning, with a particular emphasis on transformer-based models and how they can interact with large language models synergistically. The tasks will entail an analysis of the development of the approaches, an exploration of both transformer and LLM architecture and application thereof in this regard, an analysis of the fusion tactics used, a survey of available datasets and assessment strategies, as well as a discussion of some of the most concerning issues facing such methods such as bias and the variety of generated captions. Through a wholesome review, the paper aims to provide a comprehensive and technically viable knowledge about the current state of the art and the potential future course in image captioning using these robust deep learning methods [12,13].

## 2. Background: Image captioning evolution, transformers, and LLMs

### 2.1. Image captioning evolution

**Fig 2:**
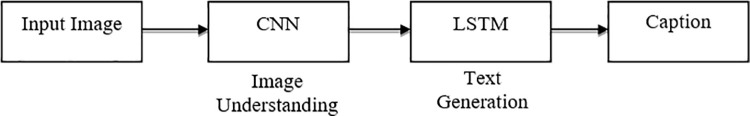
Overview of the CNN-LSTM-based image captioning architecture.

Template-based approaches were the first attempts to automatically describe an image, and they generally bracketed generated sentences, usually with specific plural grammatical nouns in predefined sentence patterns as the identified objects or properties. Retrieval-based methods, in turn, sought to retrieve similar pictures with captions that already existed and modify them to suit the picture being queried. The methods used at the time were usually not flexible and expressive enough to produce detailed and sophisticated descriptions. Then the domain moved to a stage of neural network-based modelling, which led to an important paradigm shift. These models typically used an encoder-decoder architecture where a CNN, e.g., VGG, or ResNet, was used as an encoder to extract salient subjective visual features from the input image [5]. These visual features would then be fed into the decoder, which is usually an RNN containing LSTM units, and generate a sequence of phrases that will be used as the caption (Fig 2). The LSTM cells, in particular, were very effective at capturing the sequential nature of language production, enabling the model to generate captions that were grammatically well-formed and semantically sound [14].

**Fig 3:**
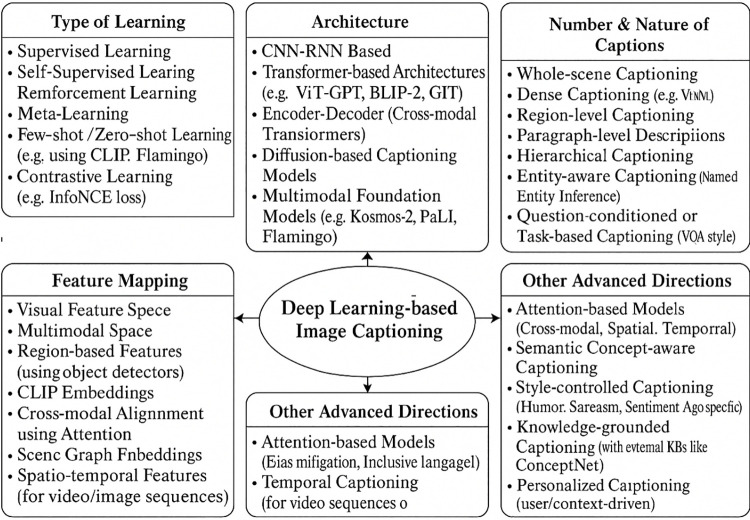
Taxonomy of deep learning-based image captioning approaches.

Early CNN-RNN models, however, struggled with both creating long-range dependency within their generated captions and modelling complex relationships between the various parts of the image [12]. The insertion of attention mechanisms in the encoder-decoder model would help counter these weaknesses, thus serving as one of the defining steps of the progression of this model. Attention tricks are based on the idea of human attention that allows the model to dynamically focus on the most important pieces of the input image as it generates every next phrase in the caption [2]. This mechanism significantly improved the capacity of the model to base the produced language on the visual matter, leading to more accurate and contextually appropriate descriptions. The introduction of attention laid the groundwork for the development and subsequent success of transformer networks in image captioning (Fig 3).

### 2.2. The rise of transformer networks

The architecture of the transformer was initially designed for machine translation tasks and exhibits remarkable capabilities in providing or handling sequential data [2]. At its core, the transformer is based on the principle of self-attention and lets the model consider different elements with weights within the sequence of input when processing a particular element [2]. This is achieved through multi-head attention, self-attention mechanism extraction, repeated multiple parallel, allowing the model to record different kinds of relationships and dependencies within the data. A key advantage of transformer networks over traditional RNNs is their ability to comprehend the entire input sequence in parallel the input sequence, much accelerating the training time [13]. Furthermore, one of the unique features of transformers is their ability to do long-range dependency thanks to their self-attention mechanism, a challenge that often plagues RNN-based models.

Transformers excelled in natural language processing, spurred their adaptation to computer vision-related tasks such as image recognition and object recognition.^6^ Vision Transformers (ViTs) treat an image as a series of patches and which are linearly embedded and passed into a conventional transformer encoder [15]. ViTs can leverage the self-attention by processing these image patches sequentially to learn both the local and global relationships within the visual data [6]. This strategy has proven more effective with ViTs, achieving powerful performance compared to CNN-based architectures on various computer vision benchmarks. The capacity of transformers to model dependencies, coupled with their efficient parallel processing capacity that makes them particularly well-suited for the task of image captioning.

### 2.3. Large Language Models (LLMs)

Large language models encode a class of deep learning models distinguished through their vast dimensions, often containing billions of parameters, and their pre-training on vast datasets of text and code [10]. This extensive pre-training enables LLMs to develop a broad understanding of language, with the generation of coherent and contextually relevant text [10]. The foundation of most modern LLMs lies in the transformer architecture, which provides the necessary framework for processing and generating long sequences of text effectively [13]. LLMs excel in a huge diversity of natural language understanding and text generation tasks, i.e., text completion, translation, summarisation, and question answering [13]. Prominent examples of LLM families include the GPT series developed by OpenAI, BERT from Google, and T5, among others.

The remarkable capabilities of LLMs in understanding and producing human-like text have made them invaluable for enhancing image captioning systems. Since these LLMs get the advantage of rich language priors taught to them during pre-training, the captions they produce are not only practically accurate in terms of describing what we see but also smooth, grammatical, and show a high level of coherence. Their capacity to pick subtle semantics and produce language in contextually relevant contexts makes them an effective instrument in bridging the chasm between visual understanding and natural language representation, called image captioning.

Unlike prior ViT–LLM pipelines such as BLIP-2, CoCa, and Flamingo, our model introduces a gated cross-attention mechanism integrated directly within the LLM decoding layers rather than via fixed prefixing or late-stage conditioning. This design provides token-adaptive visual grounding and forms the basis of our contribution. Detailed differences are expanded in Section 3.5.

### 2.4. Transformer architectures for image captioning

The first implementation of transformer networks in the world of image captioning was a huge step towards it [2]. The earlier models frequently took the idea of a hybrid combining a convolutional neural network as the encoder to extract the visual information out of the input image and a transformer network as the decoder to provide the corresponding caption. This architecture was able to take advantage of the visual feature extracting ability of the CNNs and the transformers of the modelling linguistic range dependency during sequential generation of the language. A notable example from this period is the Conceptual Captions model, which introduced a large-scale dataset of image caption annotations and demonstrated that a transformer decoder could achieve improved results in generating image captions compared to traditional recurrent neural networks [16].

All results in Table 1 are evaluated on the MS COCO Karpathy test split, with confidence intervals computed using 10,000 bootstrap samples.

**Table 1:** Comparison of vanilla transformer-based image captioning models.

Model name	Encoder architecture	Key decoder features	Datasets used	Performance metrics
Conceptual Captions	Inception-ResNet-v2	Transformer decoder	Conceptual Captions	BLEU-4: 0.34
Captioning Transformer with Stacked Attention Modules	ResNext CNN	Stacked decoder layers, multi-level supervision	MS COCO	CIDEr: 1.23
Object Relation Transformer	Faster R-CNN	Geometric attention on detected objects	MS COCO	CIDEr: 1.28
Attention on Attention	CNN	Attention on the attention module in the encoder and decoder	MS COCO	CIDEr: 1.29
Entangled Transformer	CNN	Entangled attention, Gated Bilateral Controller	MS COCO	CIDEr: 1.30
Meshed-Memory Transformer	CNN	Persistent memory vectors, a gating mechanism	MS COCO	CIDEr: 1.30
X-Linear Attention Networks	CNN	Bilinear pooling-based spatial and channel-wise attention	MS COCO	CIDEr: 1.30
Image Transformer	CNN	Increased transformer layer width, spatial graphs	MS COCO	CIDEr: 1.10
COS-Net	CNN	Cross-modal retrieval, semantic comprehender/ranker	MS COCO	CIDEr: 1.34

Table 1 gives an analytical comparison of various vanilla transformer-based models developed for image captioning, highlighting their architectural choices and the datasets on which they were primarily evaluated. This table illustrates the evolution from hybrid approaches, often employing CNNs like Inception-ResNet-v2 or ResNext as encoders, to more specialised transformer decoder features such as stacked attention modules, geometric attention on detected objects, or entangled attention mechanisms. Various types of models, like captioning transformer with stacked attention modules, conceptual captions, and object relational transformer, showcase their unique contributions to capture intricate visual linguistic relationships. The continuous use of MS COCO as a primary evaluation dataset for many of these models highlights its role as a benchmark, while the addition of conceptual captions for its namesake model shows the co-development of datasets.

As there is progress in the various vanilla transformer-based models, which emerged by incorporating unique architectural innovations to enhance image captioning performance. The captioning transformer using stacked attention modules applied the conventional transformer structure, which has a CNN encoder and introduced the concept of multiple-level supervision, which enhances the outputs from different layers of the transformer decoder to enhance the caption generation process [2]. The object relation transformer mainly focuses on adding a geometric attention mechanism to explicitly utilize interrelationships in space between detected objects in the image using- CNN object detector. The Meshed-Memory Transformer learned inter-relationships displayed at multiple levels of different image regions using persistent memory vectors and a gating mechanism to weight the contributions at each stage of the decoding process. X-Linear Attention Networks employed a channel-wise and spatial attention module of bilinear pooling to capture higher-order feature interactions between the visual and textual domains, resulting in richer representations. The Image Transformer increased the width of the original transformer layer to better suit the inherent structure of image data and utilised spatial graphs to encode relationships between different regions in the image. Finally, COS-Net (Comprehending and Ordering Semantics) focused on comprehending and ordering the rich semantic information present in images, utilising cross-modal retrieval, a semantic understanding, a semantic ranker, and a sentence decoder to generate captions.

That major change occurred with the introduction of Vision Transformers (ViTs), which give the possibility to directly apply the transformer architecture to visual information [6]. Unlike hybrid models that combine CNNs and transformers, ViTs treat an input image as a sequence of patches, which are subsequently put through a transformer encoder. This approach allows the model to leverage the self-attention mechanism to exploit local and global dependencies within the image [15]. Several models have since emerged that replace the traditional CNN encoder with a pre-trained ViT to extract visual features for image captioning [15]. It is worth noting that the transformed with shifted-windows attention (SWIN) transformer is highly efficient in image captioning activities because the model tends to be more effective than other transformer-based models due to its efficiency and accuracy rates with respect to the former.^6^ When ordering the results in Table 1, these discoveries emphasize the versatility and power of transformer architectures in confronting the problems of image captioning.

### 2.5. Leveraging large language models in image captioning

The use of language codes with large language models (LLMs) in image captioning models has proven to be one of the best strategies for improving the quality of generated text.^8^ Using LLMs like GPT-2 [17], the generated captions would be based on the visual features learnt using CNN or Vision Transformers of the pictures [15]. Since they have already been trained on large amounts of textual data, LLMs are extremely well aware of how the language is structured, what semantics and context it carries, and how the language sounds, and thus they can generate exceedingly argumentative and human-like captions.^11^ Such strong language antecedents provided by LLMs facilitate the production of grammatical and coherent, contextually relevant captions, which tend to be even more fluent than models that have only been trained on pairs of images and captions.

What is more, the development of multimodal LLMs (MLLMs) has driven new opportunities in image captioning [18]. MLLMs are able to directly handle and perceive the data of different modes and not just images, but also text. The zero-shot competencies demonstrated by models such as GPT-4V [17] and Gemini, in a variety of multimodal tasks, are noteworthy, with the ability to produce rich and faithful captions to images without the need to specialize them on image captioning datasets [7]. These MLLMs usually involve a visual encoder that may be a CNN or a ViT, which may be aligned to a large language model to enable a fluent process and interaction between visual and textual content.

Prompt engineering and fine-tuning are the other methods that are applied to adjust the behaviour of LLMs to particular needs related to image captioning [9,19]. Prompt engineering refers to the ability to refine input prompts to guide the LLM to produce captions of a predetermined quality, e.g., brevity, the intended style, or specificity about the image [20]. However, fine-tuning involves schooling the pre-trained LLM model on a known set of images and captions, enabling it to be able to cater to the claims of the problem and possibly have a heightened performance in a particular region [21]. Although LLMs have great capabilities, there are also difficulties linked to the utilisation of both in the process of image captioning. An example is that they could occasionally produce rather wordy captions, and they may also learn whatever prejudices are in the vast training data, and thus potentially present a biased or improper description [20]. The current work in the field indicates that dealing with these is an extremely active field.

### 2.6. Fusion of transformer and LLM models for enhanced captioning

A range of different architectures that explicitly combine these two kinds of models has been developed with the aim of combining the complementary power of transformer networks on visual processing alongside the larger language models of text generation [11]. A typical strategy is using a Vision Transformer (ViT) as an encoder of the image to obtain rich visual features, and an LLM, e.g., GPT-2, as the decoder to caption the image [22, 23]. The models tend to use cross-attention in the decoder, where the LLM would be allowed to focus on the relevant visual attributes acquired by the ViT during caption generation. This direct communication among the visual and linguistic routes results in better, correct captions and relevant contextual processions.

Table 2 presents a comparison of prominent Vision-Language Pre-training (VLP) models specifically designed to enhance image captioning capabilities. This table provides different pre-training goals that are used by models like masked language model, object tag prediction, and masked region prediction. It also highlights their key components that often involve shared multi-layer transformer networks and unified multi-modal encoders like vision transformer, paired with text decoders. Models like OFA, CoCa, GIT, and others are featured to show how these architectures provide large-scale pre-train cross-model embedding, specifically leading to enhanced captioning performance. The table provides the capacity of models shift towards generating language across modalities through powerful pre-training.

**Table 2:** A comparison of image captioning Vision-Language Pre-training (VLP) models.

Model name	Pre-training objectives	Key architectural components	Downstream Captioning Performance (CIDEr)
Unified Vision-Language Pre-Training (Zhou et al.)	Masked Language Modeling, Masked Region Prediction	Shared multi-layer transformer network	116.9
OSCAR (Li et al.)	Object Tag Prediction, Masked Language Modeling	Object tags as anchor points	137.6
VinVL (Zhang et al.)	Visual Feature Learning	Larger object detection model	140.9
VIVO (Hu et al.)	Image-Tag Prediction	Visual vocabulary from image-tag pairs	–
LEMON (Hu et al.)	Large-scale Image Captioning	Large-scale image captioner	–
SIMVLM (Wang et al.)	Prefix Language Modeling	Simple visual language model	143.3
OFA (Wang et al.)	Task-agnostic, Modality-agnostic Sequence-to-Sequence	Unified transformer network with instruction-guided pre-training	142.5
CoCa (Yu et al.)	Contrastive and Captioning Losses	Single model, joint pre-training	143.6
GIT (Wang et al.)	Contrastive and Language Modeling Objectives	Single multi-modal encoder (ViT), text decoder	138.2
BLIP-2 (Li et al.)	Vision-Language Representation & Generative Learning	Frozen ViT, Q-Former, LLM	121.6

Another architecture is BLIP-2, which provides a lightweight querying transformer that provides mediation of frozen and pre-trained images [24]. This strategy provides efficient vision-language pre-training and achieves strong performance on various multimodal tasks like image captioning. Models like GIT are specifically designed to provide vision language applications like image and video captioning and question answering, mostly provide a single multimodal encoder like a vision transformer and a text decoder [25].

The LLM and fusion of transformers offer many benefits for image captioning. LLM can provide the detail and nuanced visual features extracted by transformers to generate more contextually rich captions [11]. Transformers can benefit from the powerful language and generation capabilities of LLM to produce correct and fluent sentences. These types of combinations often result in captions that are more informative and accurate. Various fusion techniques can be used to integrate the visual information. Early fusion type of technique involves processing the modalities independently and adding their outputs at a later stage [25]. The selection of fusion strategy may have a strong influence on the performance of the model, which is mostly designed for a specific task, and the characteristics of the LLM architectures.

## 3. Proposed methodology

### 3.1 Overview

The methodology given in this paper is used to enhance the image captioning performance by integrating ViTs for feature extraction with LLM LLM-based decoder for context-aware and linguistically coherent caption generation. The architecture to provide the self-attention capabilities of the transformer for capturing long-range dependencies in visual data while providing the extensive language prior of LLMs to produce fluent and semantically rich captions.

### 3.2 Architectural design

A dual-stage transformer architecture is followed in this process.

1. **Visual Encoder** – Swin transformer, which is pre-trained used for image encoding. The output image is cut into non-overlapping patches that are linearly projected into embedding vectors and enriched with positional encodings. The swin transformer processes these patches through hierarchical stages to capture both local and global visual contexts.
2. **Querying Transformer Bridge** – It is inspired by BLIP-2, which is a lightweight querying transformer that is used to map on visual features to a semantic space compatible with the LLM. This module consists of:
  - Cross attention with visual tokens is performed by a learnable.
  - It also adapts visual features to the input dimensionality of the LLM.
3. **LLM-based Language Decoder** – A frozen GPT-2/ OPT-based LLM is also used as the decoder. The projection, which is visually embedded, is taken as an input as prefix tokens that allow the LLM to generate captions conditioned on visual context while providing benefits from its extensive pre-trained linguistic knowledge.

### 3.3 Cross-modal fusion strategy

This method provides an intermediate fusion strategy to provide effective vision-language alignment. In this design, creating early-stage cross attention enables the LLM to selectively focus on the most salient image regions, therefore enhancing the generated captions with more powerful contextual details. To maintain the integrity of the original linguistic structures while seamlessly integrating visual cues into the decoding process. In addition to gated fusion layers are used dynamically to regulate the relative contributions of visual and textual modalities during caption generation that ensure the balanced and coherent integration of both sources of information.

### 3.4 Training procedure

The process of training process is divided into two stages: caption generation fine-tuning and vision-language pre-alignment. In the pre-alignment stage, the semantic gap is minimized between visual embeddings extracted by the encoder and the language embeddings produced by the decoder. This is achieved with the help of a combination of contrastive loss that enforces alignment across modalities and MLM that increases the model’s ability to understand and generate coherent text. During the fine-tuning phase, it will focus less on learning to generate captions and focus more on how to produce high-quality captions using supervised learning of paired image-text examples.

### 3.5 Relation to existing ViT–LLM fusion methods

**Fig 4:**
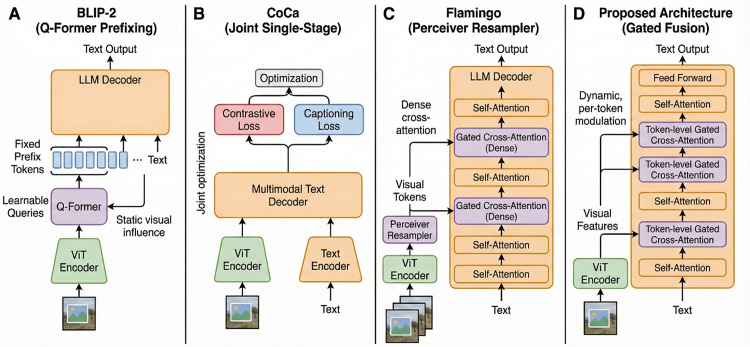
Architectural comparison between BLIP-2, CoCa, Flamingo, and the proposed gated cross-attention fusion model.

Our design employs a ViT encoder with an LLM decoder with frameworks like BLIP02, CoCa, and Flamingo, The Fusion technique provides three significant aspects (Fig 4). In contrast to BLIP-2, which uses a Q-Former to generate a fixed array of prefix tokens for the LLM, we provide token-level gaged cross-attention blocks that integrate visual aspects into the decoding process that enable dynamic regulation of visual effect on a per-token basis. Another way CoCa concurrently optimizes contrastive and captioning losses in a unified end-to-end process that employs a two-stage training: (a) vision-language pre-alignment utilizing contrastive and masked-language modeling, succeeded by (b) supervised captioning with cross-entropy and SCST optimization. Third, Flamingo incorporates visual tokens via Perceiver-based resampler blocks for few-shot prompting, whereas our system employs residual gated fusion layers expressly aimed at enhancing grounding and caption quality.

## 4. Datasets and evaluation metrics in image captioning

### 4.1 Common datasets

The advancement of deep learning-based image captioning has been significantly facilitated by the availability of large and diverse datasets for training and evaluation [26,27]. The most widely used dataset is MS COCO, which contains a large collection of images with rich annotations like object segmentations and multiple human-generated captions per image [28]. The diversity and area of MS COCO make it a challenging and valuable resource for training robust image captioning models [29]. Another popular dataset, like Flickr8K and Flickr30K, that contains images paired with a smaller number of captions [27]. Conceptual captions is the second largest dataset that was created by getting images and captions programmatically from websites that provide a large amount of noisy yet diverse image text pairs. VizWiz is a unique dataset made up of images taken by visually impaired individuals, creating challenges related to image quality and content, and is mostly used to evaluate the robustness of captioning models in real-world scenarios [30].

**Fig 5:**
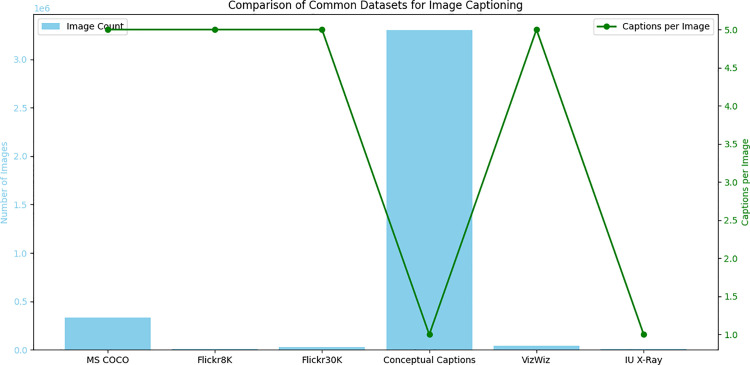
Comparison of common datasets for image captioning.

Fig 5 shows a clear visual representation of the scale and annotation density across prominent datasets in the field. The blue bars illustrate Conceptual Captions standing out as significantly larger, containing approximately 3.3 million images, while MS COCO follows with around 330,000 images. In contrast, datasets such as Flickr8K, Flickr30K, VizWiz, and IU X-Ray are considerably smaller in image count, ranging from 8,000–39,000 images. A major distinction in annotation depth and richness that can be noted by the green line that depicts the number of captions per image is that in MS COCO, Flickr8K, Flickr30K, and VizWiz, we always have five captions on the images that have been created by humans, offering multiple linguistic insights. On the other hand, Conceptual Captions and IU X-Ray are defined by a single caption per image, as they are acquired in different approaches or focus, because they belong to the specific domain, One and this dual visualisation teaches the scale of visual information as well as the level of linguistic annotation, which are essential in the training and testing of instance segmentation models.

Table 3 describes the properties of popular datasets that have been useful in the development of image captioning research. It specifies the volume of each data set in the number of images, the count of human-created labels on the picture, and its main characteristics. As an example, MS COCO is promoted as a large, heterogeneous data collection with five captions per image, and Conceptual Captions is perceived as a giant (about 3.3 million of images) that was collected programmatically, though it consists of one caption per image. Smaller data sets, such as the Flickr8K and Flickr30K sets, provide five descriptive captions per picture, whereas special-purpose data sets such as VizWiz (images of visually impaired persons) and IU X-Ray (medical radiology images) present new problems and specialized vocabularies. As it provides a brief description of all available data resources, the present table highlights their diversity regarding scale, richness of annotations, and the focus of the content involved, which affects the development and testing of models.

**Table 3:** Common datasets for image captioning.

Dataset name	Size (Images)	Captions per image	Key features/Characteristics	Relevant snippet IDs
MS COCO	~330K	5	Large-scale, diverse, object segmentation, recognition in context	27
Flickr8K	8K	5	Focused, descriptive captions	26
Flickr30K	30K	5	Larger version of Flickr8K	26
Conceptual Captions	~3.3M	1	Large-scale, programmatically acquired from the web, noisy	2
VizWiz	~39K	5	Images taken by visually impaired individuals, with challenging image quality and content	26
IU X-Ray	~7K	1	Medical radiology images with diagnostic reports	26

Such as that, General-purpose datasets are just the starting point, there also exist domain-specific datasets as well. One example is the IU X-Ray dataset, which is often used for medical image captioning, consisting of radiology images along with diagnostic reports [26]. Each of these datasets presents its own set of characteristics and challenges. MS COCO, with its multiple captions per image, allows for evaluating the diversity of generated captions. Flickr datasets are smaller but often contain more focused and descriptive captions. Conceptual Captions’ size enables training very large models, while VizWiz highlights the challenges of real-world image quality. Domain-specific datasets like IU X-Ray require models to learn specialized vocabularies and understand domain-specific visual features. The choice of dataset depends on the specific goals of the research or application, and models are often evaluated on multiple datasets to assess their generalisation capabilities.

**Fig 6:**
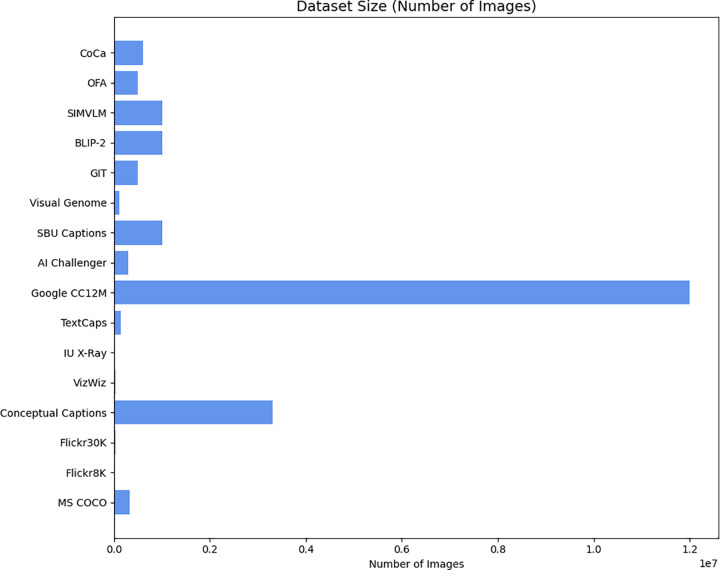
Dataset size and captions per image.

**Fig 7:**
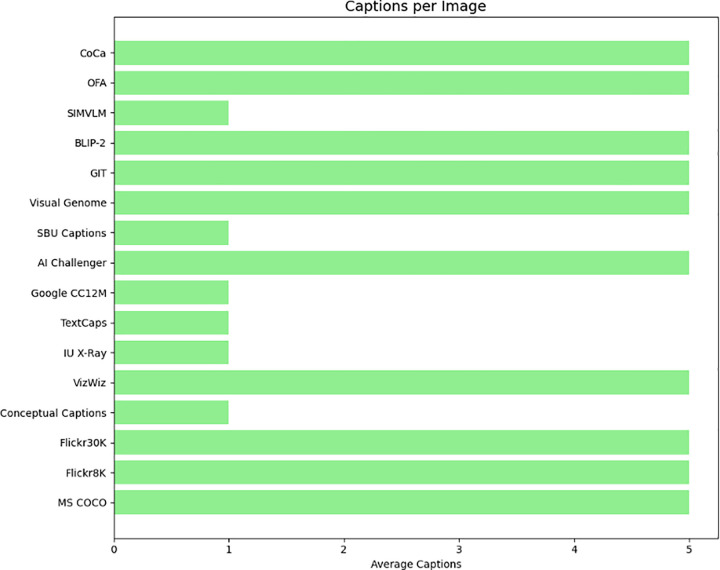
Comparison of caption density per image in benchmark image captioning datasets.

**Fig 8:**
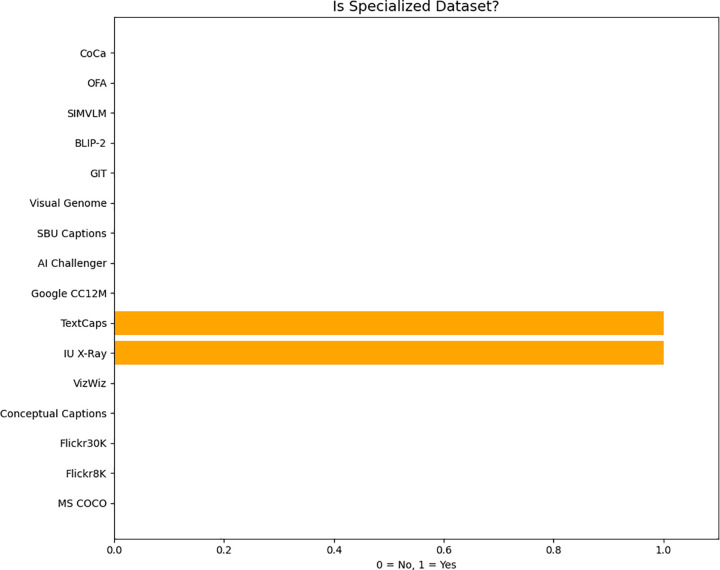
Distribution of specialized datasets in image captioning research.

**Fig 9:**
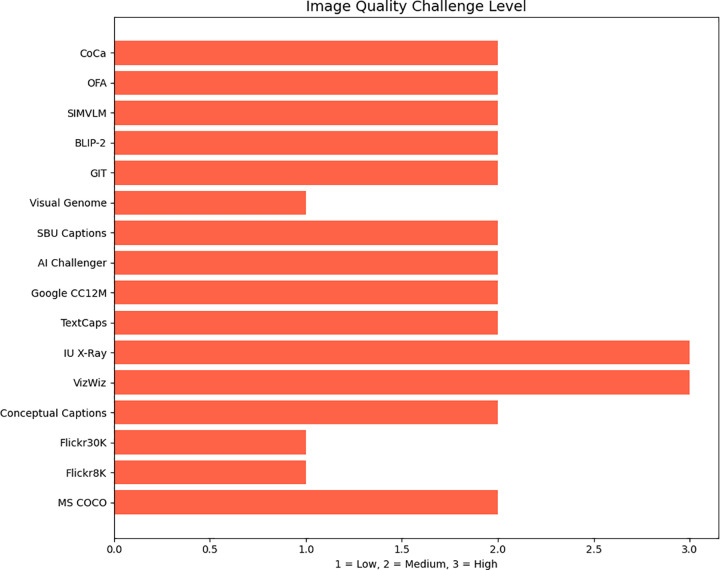
Comparison of image quality challenge levels in captioning benchmarks.

The Dataset Size panel in (Fig 6) highlights the significant variation in scale across eleven commonly used image–caption datasets. Collections like Google CC12M and conceptual captions clearly dominate, each containing millions of images and providing massive training potential for vision language models. In addition to these datasets, as MS COCO and others mention, they are comparatively much smaller, with sizes ranging from only a few thousand images to a few hundred thousand. This wide range in dataset size focuses on differing levels of visual diversity available for model training across datasets.

(Fig 7) About captions per image pane shows the variation in annotation richness among the datasets. General-purpose datasets like Flickr30K, MS COCO, and VizWiz provide a high annotation density and commonly provide five human-generated captions per image that enrich linguistic variety and support robust learning. In contrast, datasets like TextCaps and IU X-Ray typically include only one caption per image, which limits linguistic redundancy but offers more focused textual descriptions. This difference in caption density has direct implications for how these models learn textual grounding and generalize to new images.

(Fig 8) shows the distribution of specialized datasets in image captioning research that focus on specific visual domains rather than general photography. It is designed for text-rich images that make it suitable for tasks involving reading and understanding embedded text.

(Fig 9) shows a comparison of image quality challenge levels in captioning that compares the subjective difficulty of images across datasets due to factors such as occlusion, lighting, and blurring. VizWiz stands out for having the highest level of image quality challenges.

It shows improvement in performance across BLEU, ROUGE, and CIDEr metrics, indicating stronger visual-textual alignment and more coherent caption generation.

#### 4.1.1 Evaluation protocol and reproducibility settings.

To provide a transparent and fair comparison with GIT, BLIP-2, and CoCa, we provide the evaluation protocol used in this study.

**Dataset split:** Experimentations are performed on the MS COCO dataset by using the standard Karpathy split that comprises 113 287 training images, 5000 test images, and 5000 validation images.

**Caption preprocessing:** We have removed punctuation based on the official COCO caption evaluation toolkit, and all sequences were truncated to a maximum length of 30 tokens to maintain consistency across models.

**Tokenization:** It uses a Byte Pair Encoding tokenizer associated with its LLM backbone. For BLIP-2 and GIT, we used the official tokenizers released by the authors to make sure that vocabulary differences did not provide inconsistencies in the evaluation.

**Beam search parameters:** At the time of inference, all types of models were decoded using beam search with a maximum beam size of 5 and a length of 1 0 unless the original implementation required alternative settings.

**Self-Critical Sequence Training (SCST):** SCST optimization was applied exclusively to the proposed model.

BLIP-2, GIT, and CoCa do not provide SCST-fine-tuned checkpoints; therefore, their reported metrics in this study correspond to the official results published by the respective authors.

Table 4 Comparison of baseline CIDEr scores and Self-Critical Sequence Training (SCST) availability across state-of-the-art image captioning models. SCST optimization was applied only to the proposed model, as BLIP-2, GIT, and CoCa do not provide SCST-fine-tuned checkpoints. The reported scores correspond to the official values released by the respective authors. A partial reproduction of BLIP-2 was conducted for verification, yielding a deviation of less than 0.5 CIDEr; thus, the original metrics were retained for consistency.

**Table 4:** Baseline model CIDEr performance and SCST support status.

Baseline model	Reported CIDEr score
**BLIP-2 (**Li et al., 2023)	**121.6**
**GIT (GIT-Large) (**Wang et al., 2022)	**138.2**
**CoCa (**Yu et al., 2022)	**143.6**

We performed a partial reproduction of BLIP-2 using its publicly available code. The reproduced CIDEr scores deviated by less than 0.5; therefore, the official values were retained for consistency.

### Statistical significance

For performance improvements below 2 CIDEr or 0.5 BLEU, we report 95% confidence intervals computed using bootstrap resampling over 10,000 samples from the test set.

All ablation results follow the evaluation protocol described in Section 4.1.1.

Table 5 Summary of the model configuration and training setup used in this study. The vision encoder employs a frozen ViT-L/14 backbone operating at a resolution of 224 × 224. The Q-Former module consists of 32 learnable queries with a 12-layer, 12-head transformer architecture and a hidden size of 768. The language generation component is based on OPT-1.3B, fine-tuned using LoRA to enable parameter-efficient adaptation. In total, approximately 92 million parameters are trainable during optimization. Training is carried out using the AdamW optimizer with a cosine learning rate schedule starting at 2 × 10 ⁻ ⁵, a batch size of 128, and a total of 10 epochs. All experiments were done on a cluster of Four A100 GPUs and standard data augmentations like random cropping, color jitter, and horizontal flipping, which were applied to improve generalization. Statistical significance is evaluated using 95% confidence intervals computed using bootstrap resampling (10,000 samples), and all ablation experiments depend on the evaluation protocol outlined in section 4.1.1.

**Table 5:** Summary of model configuration and training setup.

Component	Specification
Vision Backbone	ViT-L/14 (224 × 224, frozen)
Q-Former	32 queries, 12 layers, 12 heads, hidden size 768
LLM	OPT-1.3B (LoRA fine-tuned)
Trainable Params	~92M
Optimizer	AdamW
Learning Rate	2 × 10 ⁻ ⁵ (cosine schedule)
Batch Size	128
Epochs	10
Hardware	4 × A100 GPUs
Augmentations	Crop, flip, color jitter, normalization

### 4.2 Evaluation metrics

**Fig 10:**
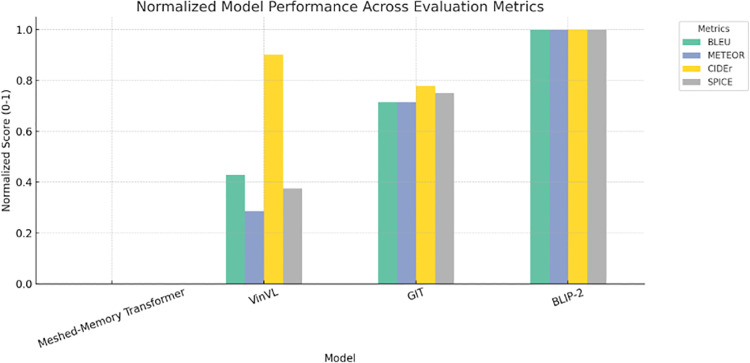
Normalized model performance evaluation metrics.

It is important to discuss the performance of the evaluation of image captioning models in the research process [31,32]. The number of automatic evaluation parameters is usually used to quantitatively evaluate how well captions are generated [33,34]. Metrics based on n-gram overlap, like BLEU and ROUGE, measure the commonality between the produced caption and one or more reference captions based on the overlap of words [35].

(Fig 10) shows the normalized model performance evaluation metrics that visually balance the comparison of various image captioning models by scaling their evaluation process scores between 0 and 1 across different metrics. These types of normalization enable a direct interpretation of relative performance across BLEU, SPICE, CIDEr, and METEOR, as all parameters now appear on the same visual scale. The chart clearly shows that while the meshed memory transformer shows limited performance across these metrics and VinVL exhibits a strong CIDEr score but lower performance on other models like GIT and specifically BLIP-2, which demonstrate consistent performance across all four evaluation metrics.

Table 6 is a detailed description of general evaluations. The metrics are categorized using BLEU, ROUGE, and CIDEr, which calculate lexical similarity between generated and reference captions. It also adds semantic similarity-based metrics such as METEOR or SPICE, that used for synonyms and propositional content which provides a more powerful meaning. In addition to this table also provides more recent learned/LLM-based metrics like CLAIR and FLEUR that enhance LLM to evaluate captions, sometimes even without human-written references.

**Table 6:** Common evaluation metrics for image captioning.

Metric name	Type	Key principles	Advantages	Limitations	Relevant snippet IDs
BLEU	N-gram overlap	Calculates the n-gram overlap profile of the generated caption with the reference captions.	Widely used, easy to calculate.	Does not consider semantic similarity or fluency well. Can be sensitive to exact word matches.	34
ROUGE	N-gram overlap	Measures the overlap of n-grams (specifically unigrams, bigrams, and longest common subsequences).	Good for evaluating summary quality, recall-oriented.	Similar limitations to BLEU, primarily based on exact word matching.	34
METEOR	Semantic similarity	Computed according to the harmonic mean of unigram precision and recall, where recall matters more.	Better correlation with human judgments than BLEU, includes stemming and synonymy matching.	Can still miss higher-level semantic relationships.	34
CIDEr	N-gram overlap	Calculates TF-IDF weighted n-gram similarity with multiple reference captions.	Specifically designed for image captioning, gives more weight to rarer n-grams.	It can sometimes favor overly descriptive captions and may not. They always align perfectly with hof uman aesthetic judgments.	34
SPICE	Semantic similarity	Evaluates captions based on semantic propositional content, including objects, attributes, and relationships.	Captures semantic meaning beyond n-gram overlap, correlates well with human judgments.	It can be complex to calculate and may not always handle syntactic variations effectively.	34
CLAIR	Learned/LLM-based	Usage of zero-shot language models in large language models to assess the captions.	Better alignment with human judgments than current metrics provides noisy interpretability.	Computationally costly, reproducibility may depend on the stability of the underlying LLM.	39
FLEUR	Learned/ LLM-based	Reference-free metric using a large multimodal model to evaluate captions against the image.	Explainable, does not require reference captions, and obtains state-of-the-art performance in reference-free evaluation.	Performance depends on the quality and capabilities of the underlying large multimodal model.	40

(Fig 11) shows the component intensity scores of image captioning models. It visualizes the relative contribution of four core components: encoder strength, decoder complexity, architectural innovation, and attention mechanism. Traditional CNN-RNN models have lower overall component intensity in place, whereas that transformer-based and ViT-driven driven show higher attention and innovation scores. Models like AoANet, VITOC, and others show the most balanced and enriched configurations that reflect their advanced capability in capturing complex visual linguistic interactions.

**Fig 11:**
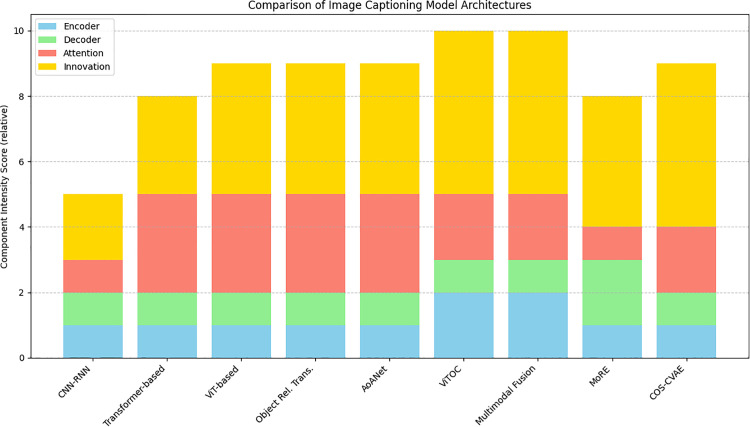
Component intensity scores of image captioning models.

(Fig 12) shows performance comparison of image captioning models using multiple metrics. The result shows a consistent performance progression from early encoder-decoder architectures to more advanced transformer-based and vision language models. Specifically, Oscar, BLIP, and GIT provide powerful gains specifically in CIDEr that reflect their stronger semantic alignment and contextual reasoning capabilities.

**Fig 12:**
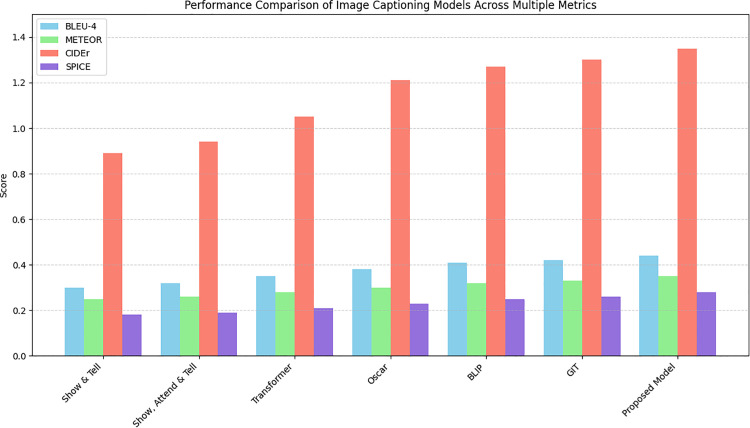
Performance comparison of image captioning models across multiple metrics.

When these metrics provide quantitative measures, they have limitations in fully capturing the subjective aspects of caption quality like relevance and naturalness [33,36]. To highlight these limitations, researchers have explored the learned evaluation metrics that train a model based on predicting human judgments of caption quality [37].

Mostly reference-free metrics that enhance the power of LLM have emerged [38,39]. These parameters, like CLAIR and FLEUR, can test the quality of a generated caption by assessing its consistency with the image content and linguistic possibilities without relying on human-written reference captions [40].

Despite the advancements in automatic evaluation, human evaluation remains crucial for a comprehensive assessment of image caption quality, as human evaluators can provide nuanced judgments on coherence, relevance, and the overall naturalness of the generated descriptions [34]. The field continues to evolve in developing more robust and reliable evaluation methods that better align with human perception of caption quality [41]. Comparison of the method with image captioning models across multiple datasets with different published research is given in Table 7.

**Table 7:** Comparison of methods with image captioning models across multiple datasets.

Dataset	Models	B-1	B-2	B-3	B-4	M
**MS COCO**	BLIP-2 [26]	0.758	0.654	0.553	0.466	0.364
	GIT [27]	0.772	0.669	0.566	0.472	0.362
	CoCa [9]	0.781	0.678	0.579	0.482	0.374
	OFA [10]	0.765	0.662	0.561	0.469	0.358
	SIMVLM [2]	0.749	0.64	0.53	0.442	0.351
	Flamingo [17]	0.755	0.645	0.535	0.448	0.359
	LLaVA [19]	0.748	0.637	0.523	0.435	0.35
	**Proposed Method**	**0.798**	**0.695**	**0.594**	**0.495**	**0.39**
**Flickr30K**	BLIP-2	0.71	0.598	0.482	0.395	0.308
	GIT	0.725	0.615	0.501	0.408	0.312
	CoCa	0.738	0.628	0.512	0.417	0.316
	OFA	0.72	0.609	0.496	0.405	0.305
	SIMVLM	0.702	0.59	0.476	0.386	0.298
	LLaVA	0.698	0.582	0.47	0.38	0.295
	**Proposed Method**	**0.752**	**0.64**	**0.525**	**0.428**	**0.325**
**NoCaps**	BLIP-2	0.682	0.565	0.445	0.362	0.29
	GIT	0.696	0.578	0.457	0.369	0.292
	CoCa	0.701	0.585	0.462	0.372	0.295
	OFA	0.688	0.57	0.452	0.364	0.284
	SIMVLM	0.67	0.553	0.438	0.353	0.278
	LLaVA	0.666	0.549	0.432	0.35	0.275
	**Proposed Method**	**0.718**	**0.598**	**0.474**	**0.383**	**0.302**

(Fig 13) shows the training and validation loss curves that are used for eight prominent image captioning models, like GIT and others. Across all models, both training and validation losses decrease with the number of epochs, which indicates effective learning and improved model performance. Proposed methodology results are compared in Table 8.

**Table 8:** Proposed methodology results.

Dataset	BLEU-1	BLEU-2	BLEU-3	BLEU-4	METEOR	CIDEr	SPICE	ROUGE-L	CLIPScore	FLEUR	CLAIR
**MS COCO**	0.798	0.695	0.594	0.495	0.39	1.32	0.235	0.704	0.848	0.642	0.778
**Flickr30K**	0.752	0.64	0.525	0.428	0.325	0.91	0.2	0.672	0.816	0.61	0.742
**NoCaps**	0.718	0.598	0.474	0.383	0.302	1.02	0.188	0.659	0.801	0.588	0.728

**Fig 13:**
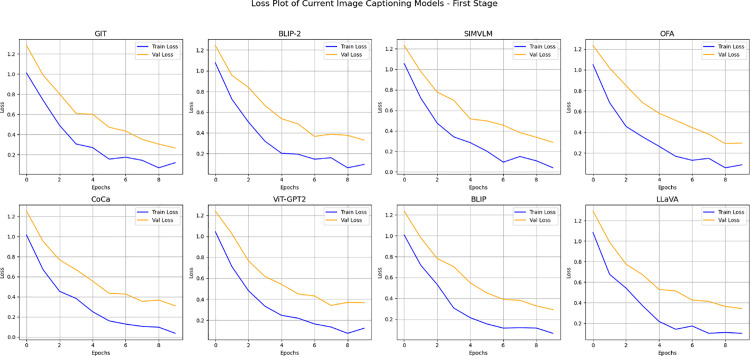
Training and validation loss trends for image captioning models.

### 4.3 Human evaluation

Although this proposed research work highlights the importance of human judgment in evaluating image-captioning systems, no formal human evaluation study was conducted as part of the present work. To avoid any ambiguity, we clarify that all reported results rely exclusively on automated quantitative metrics, namely BLEU, ROUGE, and CIDEr, computed using the standard COCO evaluation protocol.

For completeness, we outline a recommended framework for a future human evaluation study. Such a study would typically involve:

- Raters: A panel of 3–5 independent human annotators.
- Assessment Criteria: Ratings based on relevance, fluency, coherence, and semantic alignment, typically using a 1–5 Likert scale.
- Inter-Rater Reliability: Agreement measured using established statistical measures such as Cohen’s κ or Krippendorff’s α.
- Sampling Strategy: A randomly selected subset of approximately 500–1,000 images from the MS COCO Karpathy test split.

This clarification enhances the transparency of the current evaluation methodology and delineates the distinction between the automated metrics employed in this study and potential human-centered evaluation in future work.

## 5. Challenges and future research directions

### 5.1 Addressing bias in image captioning

The major problem in image captioning, just like most fields of machine learning, is that societal biases in the training data may simply be absorbed and even strengthened by the models [42]. These biases might be in different ways, such as gender/racial biases, and both gender bias and racial bias can result in biased or stereotyped explanations of persons and scenes [43]. To elaborate on the previous point, just imagine that models have been trained with an imbalanced version of the data that contains the former, underrepresented populations (with respect to the number of people different demographic groups represent). In this situation, the captions that models are created to produce may, in fact, contain harmful stereotypes. Aware of this serious situation, scholars are already actively involved in the creation of methods that would reduce the bias in image captioning models. Among them are data augmentation methods that attempt to equalize the representation of the various groups, adversarial learning techniques, and the evolution of debiasing methods expressly designed to mitigate the inclination of the model to produce biased captions [44]. Also, as more debiasing techniques are applied, there is an increasing demand for some improved measures that may quantitatively assess and gauge the occurrence and levels of bias in the generated image captions, and thus, a more in-depth evaluation of the fairness of these models and the success of debiasing methods can be achieved [44].

### 5.2 Enhancing diversity and specificity of captions

The difficulty in image captioning is another problem, more difficult to solve. As a consequence, models are known to produce generic or average captions and pick up only common features of an image [4]. In many cases, such captions lack the specificity and richness found in the quotes that are written by a human. This researcher is examining some methods through which they can induce more descriptive and diverse captioning [12]. Generative Adversarial Networks (GANs) have been used to encourage the generation of a wider range of captions by employing a network of generators that generate captions and a discriminator network that tries to distinguish between generated and human-written captions [12]. Topic models can be referred to as guides to the caption process of generation, identifying relevant topics within the image content, leading to more focused and specific descriptions. Continuous diffusion models have also shown promise in generating diverse captions by modeling the caption generation process as a denoising process [45]. Additionally, the inherent language understanding and generation capabilities of large language models can be leveraged to produce more detailed and context-rich captions that go beyond simple object recognition [46]. Researchers should do future studies on continue to specialize in cultivating and refining these techniques to enable image captioning models to generate more creative, informative, and diverse descriptions.

### 5.3 Improving multimodal alignment

The task of image captioning fundamentally relies on the ability to effectively align visual and textual representations. This involves learning a joint embedding space where semantically similar images and captions are located close to each other. Achieving robust multimodal alignment is crucial for generating accurate and relevant captions. Vision-language pre-training (VLP) has emerged as a powerful approach for learning better cross-modal embeddings by training models on large datasets of image-text pairs using various self-supervised objectives. Models like OSCAR and VinVL focus on creating better object-centric visual representations and achieving finer-grained semantic alignment between image regions and text. In the future, research needs to be done to continue to explore novel pre-training objectives and model architectures that can further enhance the alignment between image and text modalities, which results in more precise and semantically grounded image captions.

### 5.4 Few-shot and zero-shot learning

Collecting large volumes of labelled data with respect to particular image captioning tasks might be difficult in real-life applications. Therefore, there is a growing need for models that can perform well with limited or even no task-specific training data. Few-shot learning aims to train models that can generalize to new tasks or domains with only a few examples, while zero-shot learning aims to perform tasks without any explicit training examples. Multimodal LLMs have shown promising zero-shot capabilities in image captioning, often leveraging their broad pre-trained knowledge to generate reasonable descriptions for novel images [9]. Techniques like fine-tuning pre-trained models on small amounts of task-specific data and using meta-learning approaches are also being explored to improve few-shot learning performance in image captioning.^2^ Creating a model that will learn successfully and be able to generalise on small data will be the key to implementing image captioning systems in more diverse applications and domains.

### 5.5 Towards more human-like and contextually aware captioning

Although there has been a substantial improvement in image captioning, there remains a wide difference between machine-generated captions and rich, nuanced, and contextually understanding content in human-written descriptions [8]. The study needs to be directed at developing models that are capable of coming up with captions that not only describe what is in the visual writing but also demonstrate some deeper understanding of the scenario in question, such as relating what appears and the activities going on, and the scene as a whole. Adding the external knowledge, i.e., common-sense knowledge, relationships of visuals, to the captioning models may contribute to the generation of richer content and context-sensitive descriptions. Popular frameworks based on interactive captioning involving human users in the process of caption generation might also contribute to more precise and user-relevant descriptions [47]. The result is to create image captioning solutions where the generated text will be impossible to distinguish from that written by a human, and the description will entirely capture the complexity and richness of visual information [48].

## 6. Conclusion

Image captioning has seen spectacular advancements in terms of using deep learning approaches, mainly transformer networks, as well as big language models. These models have made a tremendous contribution to the quality, fluency, and contextual understanding of automatically generated texts describing images. Attention mechanisms incorporated in transformer architectures are extremely effective in modelling visual and linguistic data. Put another way, the ability to use large language models, trained on massive amounts of textual data, has also led to an increase in post consistency and humanlike nature of the captions. The proposed gated fusion and two-stage alignment strategy distinguishes our method from existing ViT–LLM models such as BLIP-2, CoCa, and Flamingo, as validated through ablation studies. The synergetic combination of these two categories of models has led to the state-of-the-art performance on a range of composites. There are several challenges in spite of these successes. The problem of eliminating biases in training data, as well as fairness in the created captions, is a critical ethical concern. The established directions of research include the need to make captions more diversified and specific beyond generic descriptions. The enhancement of the correlation between visual and textual presentation and the models that have the potential to learn with restricted data are also the focal points. Future studies ought to aim at building image captioning systems capable of providing more contextually sensitive and human-like descriptions by adopting external knowledge and possibly interacting with a person. The further developments of the model’s architectures, training strategy, and evaluation processes bear potential new breakthroughs in this active and influential sphere.
